# Organometallic Synthesis of Platinum-Based Nanomaterials for the Oxygen Reduction Reaction

**DOI:** 10.3390/nano16060364

**Published:** 2026-03-17

**Authors:** Nargiz Kazimova, Nuria Romero, Jérôme Esvan, Marjorie Cavarroc, Sara Cavaliere, Karine Philippot

**Affiliations:** 1 CNRS, LCC (Laboratoire de Chimie de Coordination), UPR8241, University of Toulouse, UPS, INPT, CEDEX 4, 31077 Toulouse, Francenuria.romero@lcc-toulouse.fr (N.R.); 2 ICGM, University Montpellier, CNRS, ENSCM, CEDEX 5, 34095 Montpellier, France; 3 CIRIMAT, Université de Toulouse, CNRS-INPT-UPS, 4 Allée Emile Monso, BP 44362, 31030 Toulouse, France; jerome.esvan@ensiacet.fr; 4 Safran-Tech, Rue des Jeunes Bois, 78772 Magny les Hameaux, France; marjorie.cavarroc@safrangroup.com

**Keywords:** carbon-supported platinum nanomaterials, organometallic synthesis, oxygen reduction reaction, electrocatalysis

## Abstract

Pt-based catalysts remain the most effective materials for the oxygen reduction reaction (ORR) at the cathode of proton exchange membrane fuel cells (PEMFCs); however, platinum scarcity and high cost severely limit the large-scale deployment of the technology. Improving catalytic activity and durability through precise control of nanoparticle morphology is therefore crucial for reducing costs and enhancing sustainability, enabling PEMFC widespread adoption. In this context, carbon-supported Pt-based nanoparticles with a 30 wt.% Pt loading were synthesized by an organometallic chemistry approach using hexadecylamine (HDA) as a stabilizer, allowing fine control over nanoparticle morphology. Two distinct synthesis pathways (one-pot and two-step procedures) were used to prepare platinum catalysts supported on KetjenBlack EC-300J (KB), and their influence on the electrocatalytic activity of the obtained nanomaterials was studied. Furthermore, the effect of HDA stabilization on catalyst performance was investigated. Directly synthesized Pt/KB catalysts exhibited similar ORR mass activity, regardless of whether or not HDA was present. Pt/KB prepared by the two-step procedure showed a significantly lower performance. Additionally, despite a larger loss of electrochemical surface area during an accelerated stress test compared to a commercial Pt/C reference, Pt^HDA^/KB and Pt/KB catalysts maintained stable mass activity and limited specific activity degradation, highlighting the beneficial effect of nanoparticle stabilization in the presence of HDA on prolonged electrocatalyst cycling.

## 1. Introduction

The depletion of fossil fuels and increasing environmental pollution have driven the urgent need for efficient, low-carbon technologies for sustainable energy conversion [1,2]. Proton exchange membrane fuel cells (PEMFCs) are among the most promising energy conversion technologies due to their high efficiency, low environmental impact, and versatility for transportation, stationary, and industrial applications [3]. However, PEMFC performance is strongly governed by electrocatalysts, particularly at the cathode, where the oxygen reduction reaction (ORR) is kinetically sluggish [4]. Platinum (Pt) remains the most effective single-metal catalyst for the oxygen reduction reaction (ORR) [5], but its scarcity in the Earth’s crust, unsustainable mining practices, and high cost limit large-scale applications. Nanostructuring of catalysts minimizes the catalytic load on PEMFC compared to bulk materials and enhances surface and active sites. The possibility of controlling the nanostructuring of Pt is therefore an attractive strategy to enhance its catalytic activity and mitigate these limitations. Pt nanoparticles provide a high surface-area-to-volume ratio and a high density of weakly coordinated, high-energy surface atoms, leading to improved electrocatalytic activity. Beyond particle size, catalyst shape and the nature of exposed crystal facets play a decisive role in ORR kinetics. Consequently, facet-dependent, shape-controlled Pt nanocrystals have emerged as a promising research direction for PEMFC ORR catalysis [6]. Recent efforts have focused on polyhedral three-dimensional architectures with tunable corner and edge truncation, enclosed by controlled proportions of low-index facets, (1 1 1), (1 1 0), and/or (1 0 0), which exhibit enhanced catalytic activity and have attracted increasing attention [7].

Conventional chemical synthesis routes such as reduction with NaBH_4_, the polyol method, or microwave-assisted synthesis have been widely used to produce Pt nanoparticles with controlled size and morphology [8,9,10,11,12]. However, these conventional wet chemical methods often lead to a limited control over surface chemistry, whereas organometallic ones allow for better control of the structural and surface characteristics of nanoparticles [4]. The organometallic approach may thus offer key advantages for ORR nanomaterials, including precise control over particle size (between 1 and 10 nm) and shape, tunable surface chemistry via stabilizing agents, and uniform dispersion on carbon supports. In addition, this method requires mild conditions of temperature (−10 °C ≤ T ≤ 150 °C) and pressure (1–3 bar of H_2_ or CO) [13,14]. Precursor decomposition into bare atoms occurs through the breaking of metal-carbon bonds or by displacement of ligands, leading to the nucleation step [14]. Then, the stabilizer and/or the support used allow control over the growth step of the nanoparticles [15]. Several works demonstrated the role of stabilizer and carbon support on the electrocatalytic properties of nanomaterials prepared by the organometallic approach [16,17,18,19].

Enhancing catalyst durability is essential for PEMFC performance and relies on both advanced catalyst architectures and optimized support materials [20]. Carbon black supports are widely used because of their high conductivity and surface area, which promote efficient electron transport, catalyst and ionomer distribution, and gas accessibility.

In this work, the solution synthesis of carbon-supported Pt nanomaterials by taking advantage of the organometallic approach are described together with their application in ORR electrocatalysis in acidic conditions. The tris(norbornene)platinum organometallic complex ([Pt(NBE)_3_]) was used as the Pt precursor, and hexadecylamine (HDA) served as a stabilizer. The obtained nanoparticles were supported on a commercially available carbon black, KetjenBlack EC-300J, by two different synthesis procedures: a direct one-pot procedure in the presence of the support, and a two-step one entailing synthesis of Pt NPs and their subsequent deposition on the support [21]. ORR activity and stability in acidic conditions were compared to those of a commercial Pt/C catalyst with the same Pt loading (30 wt.%) as a reference. The obtained results provide promising insights for future development of PEMFCs catalysts with defined structure and morphology.

## 2. Materials and Methods

All chemical syntheses were carried out using Fisher-Porter reactors, a glove box (MBraun, <1 ppm H_2_O, <1 mmol O_2_) and argon-vacuum line techniques. Ar (≥99.999%, O_2_: ≤2 ppm, H_2_O: ≤2 ppm, C*_n_*H*_m_*: ≤0.5 ppm, CO: ≤1 ppm, CO_2_: ≤1 ppm) and H_2_ (≥99.999%, O_2_: ≤2 ppm, H_2_O: ≤2 ppm, C*_n_*H*_m_*: ≤0.5 ppm, CO: ≤0.5 ppm, CO_2_: ≤0.5 ppm) were purchased from Air Liquide (France). Solvents were purified using MBraun SPS-800 solvent purification equipment (MBraun, France) and degassed by the freeze-pump-thaw method before use. [Pt(NBE)_3_] was purchased from NanoMePS company in Toulouse, France. Hexadecylamine (HDA), perchloric acid (HClO_4_, 70%, suprapur^®^ Sigma Aldrich Chimie, St Quantin Fallavier, France and Nafion^®^ ~5 dispersion in low aliphatic alcohols (Ion Power Gmbh, Munich, Germany) and water were purchased from Sigma-Aldrich (France). All these reactants were used as received. KetjenBlack EC-300J carbon was purchased from Fuel Cell Store (Bryan, Texas, USA). A reference commercial 30 wt.% Pt/C catalyst was purchased from Johnson Matthey (France).

**Transmission Electron microscopy (TEM) and High-Resolution Transmission Electron Microscopy (HR-TEM) analyses.** TEM and HR-TEM studies were performed at the Raimond Castaing Microcharacterization Centre, CNRS-UAR 3623, Toulouse. TEM images were acquired using JEOL JEM 1400 operating at 120 kV with a point resolution of 2.0 Å. HR-TEM images were acquired using JEOL JEM-ARM200F Cold FEG operating at 200 kV with a point resolution of >1.9 Å. Preparation of samples for analysis consisted in drop-casting one drop of each suspension of NPs in toluene onto a carbon-coated copper grid. Before introducing the grids to the microscope chamber, they were dried under a vacuum for one day. Particle mean size and size distribution were obtained through manual measurement of at least 100 particles, using ImageJ software (1.54p).

**Atomic Force Microscopy (AFM)*****.*** A SmartsSPM-1000, AIST-NT microscope was used to investigate the surface morphology of Pt nanomaterials deposited on a silicon substrate. Topography was measured in tapping mode with a cantilever with a spring constant k = 40 N/m, and a silicon tip with 10 nm radius of curvature. AFM images (512 × 512 pixels) were obtained on various scan areas such as 2 × 2 µm and 20 × 20 µm, for example.

**Inductively Coupled Plasma Optical Emission Spectroscopy (ICP-AES)*****.*** Platinum contents were determined by inductively coupled plasma atomic emission spectroscopy using a Thermo Scientific ICAP 6300 instrument. Pt nanomaterials were dissolved in HCl:HNO_3_ (3:1 *v*/*v*) mixture and diluted with MilliQ water.

**Fourier Transform InfraRed spectroscopy (FT-IR)*****.*** Spectra were recorded on a PerkinElmer GX2000 spectrometer inside a glove box on solid samples. Data were provided in wavenumber (cm^−1^). The sample preparation was as follows: The collected supernatants resulting from the purification of the nanoparticles by pentane washings were mixed and then vacuum-dried, leading to a solid residue, which was deposited on the sample holder for FT-IR measurement. FT-IR measurements of Pt nanoparticles were performed on the powder obtained after purification. The spectra of the commercial compounds were measured as received.

**Electrochemical measurements*****.*** The ORR performance of prepared Pt-based nanomaterials was investigated using the rotating disk electrode (RDE) technique. The experiments were carried out in a 3-electrode cell connected to an electrochemical workstation BioLogic Potentiostat SP-300. The reference electrode was a reversible hydrogen electrode (RHE), while the counter electrode was a graphitic rod. The glassy carbon electrode (working electrode, geometric area, A_geo_, 0.196 cm^2^) was polished to a mirror finish with alumina slurries and sonicated in isopropanol and Milli-Q water to remove polishing residues. The catalyst inks were prepared using an adjusted quantity of each Pt nanomaterial as the function of their Pt loading (Pt wt.%) that was dissolved in 1 mL of isopropanol in addition to 20 µL of 5% *v*/*v* Nafion^®^ (dispersed in lower aliphatic alcohols and water) and 3.98 mL of Milli-Q water. The total volume (V_total ink_) of each catalyst ink was 5 mL. Each catalyst ink was sonicated for 45 min, then 20 µL (V_deposited ink_) were deposited on the glassy carbon electrode followed by drying via rotation of the electrode at 350 rpm. Catalyst loading resulted in 20 µg^Pt^ cm^−2^_geo_ (L_pt_). The same protocol was applied with the reference commercial 30 wt.% Pt/C catalyst.

**ECSA Calculation**

To activate the catalyst surface, 50 cycles of cyclic voltammetry (CV) from 0.05 V to 1.05 V vs. the RHE were performed at 50 mV s^−1^ in N_2_ saturated 0.1 M HClO_4_ electrolyte for each sample. Three cycles were performed at *v* = 20 mV s^−1^ from 0.05 V to 1 V in N_2_ saturated 0.1 M HClO_4_ to assess the Pt electrochemical surface area (ECSA) through the hydrogen desorption peak (Equation (1)) [22].


(1) $$Q(\mu C)=\frac{1}{v}\int_{0.05}^{0.4}idE$$

The theoretical value of Q_0_ (H_upd_) = 210 µC cm_pt_^−2^was taken for platinum surface (S_pt_) calculations according to Equation (2):


(2) $$S_{pt}(cm_{pt}^{2})=\frac{Q}{Q_{0}}$$

The ECSA was calculated by normalizing the S_pt_ to Pt loading (L_pt_ = 20 µg_pt_ cm_geo_^−2^) and geometric surface area of the glassy carbon electrode (A_geo_ = 0.196 cm_geo_^2^) (Equation (3)).

(3) $$ECSA(m_{pt}^{2}g_{pt}^{-1})=\frac{S_{pt}}{L_{pt}\cdot A_{geo}}$$

**ORR Activity Evaluation**

The ORR polarization curves were recorded by linear sweep voltammetry (LSV), from 0.05 to 1.05 V at 20 mV s^−1^, in O_2_ saturated 0.1 M HClO_4_. The background LSV in N_2_-saturared 0.1 M HClO_4_ was subtracted from the ORR curves and normalized by the geometric area, A_geo_ (Equation (4)).

(4) $$j(mA\;{cm}_{geo}^{-2})=\frac{i_{O2}-i_{N2}}{A_{geo}}$$

The Koutecky–Levich equation (Equation (5)) was used to calculate kinetic current (i_k_), where diffusion limiting current (i_dif_) was taken at 0.4 V vs. the RHE:

(5) $$i_{k}(mA)=\frac{i_{dif}\cdot i_{T}}{i_{dif}-i_{T}}.$$

The ORR specific activity (I_s_) was calculated from the normalization of i_k_ to the S_Pt_ (Equation (6)), and mass activity (I_m_) was derived from the normalization of i_k_ to the Pt loading L_pt_ at 0.9 V *vs.* the RHE, under kinetic control (Equation (7)) [23].

(6) $$I_{s}(mA\;{cm}_{pt}^{-2})=\frac{i_{k}}{S_{pt}}$$

(7) $$I_{m}(A\;{mg}_{pt}^{-1})=\frac{i_{k}}{L_{pt}\cdot A_{geo}}$$

Theoretical ECSA calculation (density of Pt, *ρ_pt_* = 21.45 g cm^−3^):

(8) $$ECSA\;(m^{2}g^{-1})=\frac{6\times 10^{3}}{\rho \cdot d_{sphere\;(nm)}},$$

(9) $$ECSA\;(m^{2}g^{-1})=\frac{6\times 10^{3}}{\rho \cdot a_{edge\;(nm)}}.$$

**Accelerated stress test (AST)*****.*** An AST was performed on down-selected catalysts and on the reference Pt/C consisting of 10,000 voltage cycles (triangular wave) between 0.6 and 0.925 V/RHE at 100 mV·s^−1^ scan rate in N_2_ saturated 0.1 M HClO_4_ electrolyte. The evolution of ECSA and ORR of the catalysts were assessed upon AST.

**Powder X-ray diffraction (XRD)**. The data were collected in transmission mode at room temperature on a PANalytical X’Pert PRO MPD powder diffractometer with Bragg–Brentano theta/theta geometry, equipped with a standard Cu X-ray tube (λ_Kα1_ = 1.54056 Å and λ_Kα2_ = 1.54439 Å). Powder samples for XRD analysis were loaded into 0.5 mm × 80 mm glass capillaries (WJM-Glas Müller GmbH, Berlin, Germany (ref. G-05-001-80)) to a height of ~2 cm. All diffractograms treatments (background removal, peaks search, and profile refinement) were conducted using the HighScore Plus software (Malvern Panalytical).

The Scherrer equation (Equation (10)) with HighScore Plus software program allowed determination of the crystallite size where D is the crystallite size in nm, λ is the wavelength of the radiation in nm (1.54 Å K_a1_ of Cu), K is the Scherrer constant which is 0.9, and β_measured_ is observed full width at half maximum (FWHM) in radian. θ is Bragg angle in radian.

(10) $$D\;(nm)=\frac{K\lambda }{\beta \times cos\;\theta }$$

**X-Ray Photoelectron Spectroscopy (XPS)**

The photoelectron emission spectra were recorded using a monochromatized Al Kα (hν = 1486.6 eV) source on a ThermoScientific K-Alpha system. The X-ray spot size was about 400 µm. The pass energy was fixed at 30 eV with a step of 0.1 eV for core levels and 160 eV for surveys with a step of 1 eV. The spectrometer energy calibration was performed using the Au 4f_7/2_ (83.9 ± 0.1 eV) and Cu 2p_3/2_ (932.8 ± 0.1 eV) photoelectron lines. XPS spectra were recorded in direct mode N (Ec) and the background signal was removed using the Shirley method. Data processing was performed using Avantage software (ThermoScientific). The flood gun was used to neutralize charge effects on the top surface. Calibration of the spectra was performed with C1s binding energy at 284.3 ± 0.1 eV. Samples were measured as synthesized and deposited onto a carbon tape before transfer into the XPS chamber.

**Synthesis of HDA-Stabilized Pt Nanomaterial (Pt/HDA)**

In a glove box, HDA (9 mg, 37 µmol) was added to a Schlenk flask together with 10 mL of toluene as solvent under Ar. Also in the glove box, [Pt(NBE)_3_] precursor (16.5 mg, 36 µmol) was introduced into a Fisher-Porter (FP) reactor. Outside of the glove box, under Ar, the toluene solution of HDA (HDA/Pt ratio is 1:1) was transferred to the FP reactor containing the Pt precursor while stirring. For syntheses performed at −10 °C, the reaction mixture was cooled by placing the FP reactor in an ice bath with salt, prior and during the application of the required hydrogen (H_2_) pressure. The FP reactor was pressurized under 3 bar of H_2_ for 10 min at −10 °C, which led the reaction mixture to become black after a few minutes. Then, the reaction mixture was kept overnight at 23 °C under vigorous stirring. After this reaction time, the H_2_ excess was removed under vacuum, and the stirring stopped to leave the reaction mixture to precipitate. After separation of the supernatant under Ar with a cannula, the black solid was washed with pentane (3 × 15 mL) to remove excess of HDA (see FT-IR data, Figure S3), before drying under a vacuum.

**Synthesis of Carbon-Supported Pt Nanomaterials**

Two procedures were used to synthesize carbon-supported Pt nanomaterials on commercial KetjenBlack-EC 300J (KB; 60 mg). The Pt loading onto the carbon support was targeted to 30 wt.%.

The first synthesis procedure (direct one-pot synthesis) was similar to that of the HDA-stabilized Pt nanomaterial, except all reagents, namely [Pt(NBE)_3_] (100 mg; 209.4 µmol), HDA (50 mg; 207.5 µmol) and KB support (60 mg) were directly added into the FP reactor with toluene (62 mL) as solvent. The reaction mixture was then pressurized under 3 bar of H_2_ for 10 min at −10 °C before keeping it overnight at 23 °C under vigorous stirring. After this reaction time, the H_2_ excess was removed under vacuum, and the stirring stopped to leave the reaction mixture to precipitate. After separation of the supernatant under argon with a cannula, the black solid was washed with pentane (3 × 100 mL) to remove excess HDA, before drying under vacuum (Pt^HDA^/KB). In addition, one KB-supported Pt nanomaterial was prepared under the same conditions except no HDA was added (Pt/KB).

The second indirect procedure involved the synthesis of Pt NPs colloidal suspension, as described above, followed by its subsequent deposition on the carbon support by transfer onto a toluene suspension of KB (Pt^HDA^/KB-I). The reaction mixture was stirred at RT for five days. The solid material was easily isolated by decantation; the supernatant was removed by cannula and the solid was washed several times with pentane to remove excess HDA and eventual unsupported Pt NPs. Three different Pt loadings were investigated, namely 30, 40 and 50 wt.%. As the 30 wt.% loading appeared to lead to the best ORR activity, only the results obtained with this nanocatalyst are described in the main text, while the others are provided in the Supplementary Materials (Figure S5, Table S1).

## 3. Results and Discussion

The olefinic tris(norbornene)platinum organometallic complex ([Pt(NBE)_3_]) was selected as the metal precursor and hexadecylamine (HDA) as the stabilizer. A key advantage of the [Pt(NBE)_3_] complex is its high reactivity under hydrogen, which enables rapid nanoparticle formation without requiring long reaction times or elevated temperatures. Furthermore, the norbornane produced during the hydrogenation of ([Pt(NBE)_3_] can be easily removed under a vacuum, resulting in the absence of unwanted byproduct that may coordinate at the nanoparticle surface [24]. Since electrocatalytic reactions occur at the surface, any contamination can block active sites and thus negatively impact activity and performance. Using a precursor that minimizes residual contamination is therefore highly advantageous for the control over surface state of the synthesized nanoparticles. However, as a stabilizer may also improve stability, HDA was selected as a stabilizer because it has previously been shown to promote the formation of small Pt nanoparticles through efficient nitrogen–metal surface interactions, while being labile enough to avoid blocking active sites [25]. In the context of a Pt surface free of undesired contaminant, we wanted to study the influence of the voluntarily added HDA on the structural and ORR properties of the obtained Pt nanomaterials [26].

**Scheme 1:**
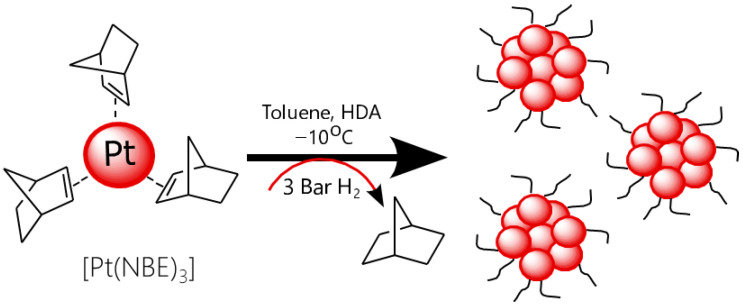
Synthesis of HDA-stabilized Pt nanoparticles from ([Pt(NBE)_3_]) complex at −10 °C in toluene, under 3 bar of H_2_.

The Pt nanoparticles synthesis was performed under mild reaction conditions (3 bar of H_2_, at −10 °C), in toluene as a solvent (Scheme 1).

### 3.1. Synthesis and Structural Characterization of Pt Nanoparticles Stabilized with HDA

**Figure 1:**
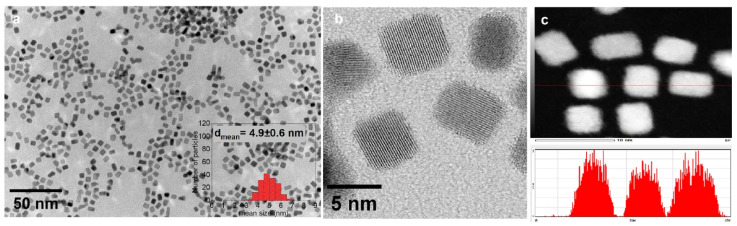
(**a**) TEM image of Pt^HDA^ NPs formed by decomposition of [Pt(NBE)_3_] in toluene under 3 bar of H_2_ in the presence of HDA as stabilizer, at −10 °C; (**b**) HR-TEM image of Pt^HDA^ NPs; (**c**) STEM-HAADF/EDX line analysis of Pt^HDA^ NPs.

Transmission electron microscopy (TEM) analysis of HDA-stabilized Pt NPs (Pt^HDA^) indicated the formation of cubic-type, crystalline, and well dispersed Pt NPs with a mean size of ca. 5 nm (Figure 1a). This demonstrates the role of hexadecylamine in selective plane growth by preferential binding to the (1 0 0) facets of platinum, which favors the formation of a cubic shape. Other capping agents have demonstrated the production of cubic morphologies for platinum (e.g., polyacrylic acid) in conventional wet chemical reduction methods [27] or reshaping approaches through a seed-mediated route of commercial quasi-spherical Pt/C [28]. High-resolution transmission electron microscopy (HR-TEM) and scanning transmission electron microscopy-high angle annular dark field (STEM-HAADF) analyses of Pt^HDA^ were performed in order to obtain more information on their crystalline structure and morphology (Figure 1a,b). The images clearly evidence the presence of well-defined, regular-shaped and crystalline Pt NPs with parallel lattice fringes. The measured interplanar spacings are consistent with metallic Pt. Additionally, EDX line mapping confirmed the presence of Pt metal all along the NPs (Figure 1c).

**Figure 2:**
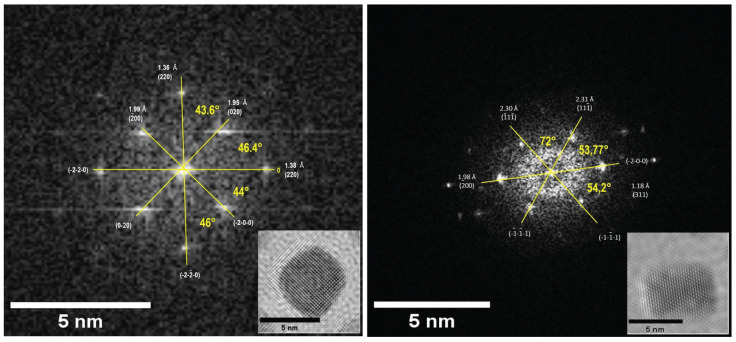
FFT analyses from HR-TEM images of two different individual Pt^HDA^ NPs.

Cube-like morphologies are typically associated with dominant (1 0 0) facets in fcc Pt; however, the Fast Fourier Transform (FFT) analysis of HR-TEM images performed on single Pt^HDA^ NPs (Figure 2) did not allow a straightforward identification of the prevailing crystallographic planes [29]. In Figure 2 (left), the obtained diffraction patterns clearly indicate the presence of the lattice planes corresponding to the (1 1 1) and (0 1 1) axes of the bulk fcc Pt crystal structure. Observed interplanar angles were found to be 43.6° between the (2 2 0) and (0 2 0) planes, 46.4° between the (0 2 0) and (2 2 0) planes, 44.0° between the (2 2 0) and (2 0 0) planes, and 46.0° between the (2 0 0) and (2 2 0) planes. In Figure 2 (right), the observed interplanar angle was 72.0° between the (1 1 1) and (1 1 1) planes, 53.7° between the (1 1 1) and (2 0 0) planes, and 54.2° between the (2 0 0) and (1 1 1) planes. These results indicate that Pt^HDA^ NPs have anisotropic strain relative to the standard fcc lattice along the (0 0 1) direction. Atomic force microscopy (AFM) confirmed the cubic or cuboid shape of the Pt nanoparticles (Figure S1). Measurement of the Z values led to an average thickness in the range 4–6 nm between XY positions, in excellent agreement with the size determined from TEM images.

**Figure 3:**
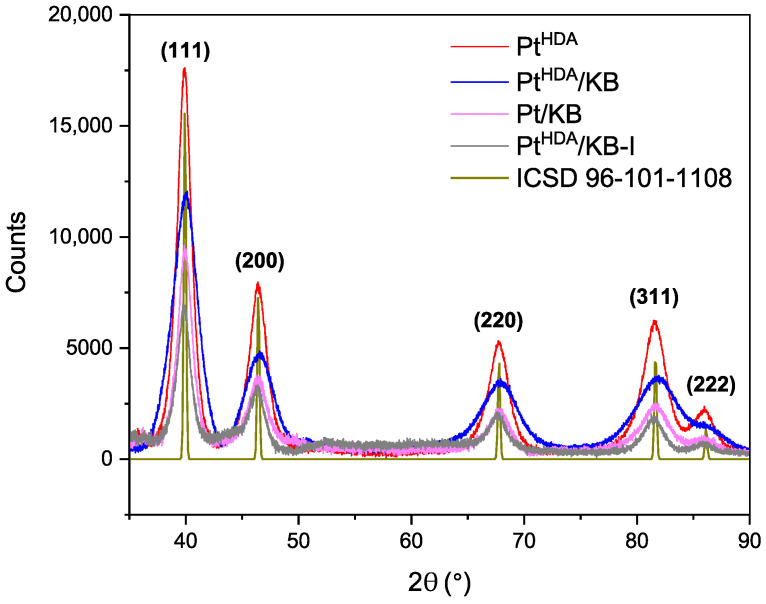
Powder-XRD patterns of Pt^HDA^, Pt^HDA^/KB, Pt/KB and Pt^HDA^/KB-I nanomaterials in comparison with the fcc Pt reference.

Powder X-ray diffraction (XRD) pattern of Pt^HDA^ NPs (Figure 3) showed the presence of well-defined peaks in agreement with those of a Pt fcc reference (ICSD 96-101-1108), confirming the Pt NPs were crystallized in the fcc phase. The Scherrer equation method was applied to determine the size of crystallites. For 2θ peaks at 67.7° (2 2 0), 46.3° (2 0 0) and 39.9° (1 1 1), mean sizes of 4.5 nm, 4.8 and 5.6 nm were found, respectively. These data align well with the average particle size measured by TEM (4.9 nm).

FT-IR spectra of purified Pt^HDA^ nanoparticles clearly showed the presence of a small residual amount of HDA at the Pt nanoparticle surface, indicating that washing with pentane was effective in removing excess HDA (see Figure S3). Such a low amount of HDA is favorable for the activity of the Pt surface atoms while maintaining the stability of the nanoparticles.

### 3.2. Synthesis and Structural Characterization of Carbon-Supported Pt Nanoparticles

Supported Pt nanoparticles were prepared using Ketjenblack EC-300J as a carbon support via two different pathways: a one-pot synthesis in the presence of the support, and a two-step synthesis based on an impregnation method.

In the one-pot (also called direct) pathway (Pt^HDA^/KB), the carbon support was introduced at the beginning of the reaction, and the synthesis was otherwise identical to that used for preparing Pt^HDA^ nanoparticles. In order to assess the influence of the support on the NP size and stabilization, another carbon-supported Pt nanomaterial was prepared using the same route but with the absence of the HDA stabilizer. For this synthesis, the [Pt(NBE)_3_] complex was simply decomposed in toluene under 3 bar of H_2_ at −10 °C in the presence of Kentjenblack EC-300J (Pt/KB) only.

For the two-step (also called indirect) pathway (Pt^HDA^/KB-I), a freshly synthetized colloidal suspension of preformed Pt^HDA^ nanoparticles was transferred onto a toluene suspension of Ketjenblack EC-300J. The reaction mixture was stirred for five days at room temperature in order to optimize the impregnation and foster the Pt nanoparticles’ deposition onto the KB support (Pt^HDA^/KB-I). In both cases, the resulting Pt/C materials were washed with pentane to remove excess HDA and dried under vacuum.

For both syntheses, a nominal Pt loading of 30 wt.% on carbon was targeted (upon an optimization study as shown in the Supplementary Materials, see Table S1 and Figure S4), and ICP-AES analysis allowed us to confirm that very close values were obtained, as indicated in Table 1.

**Figure 4:**
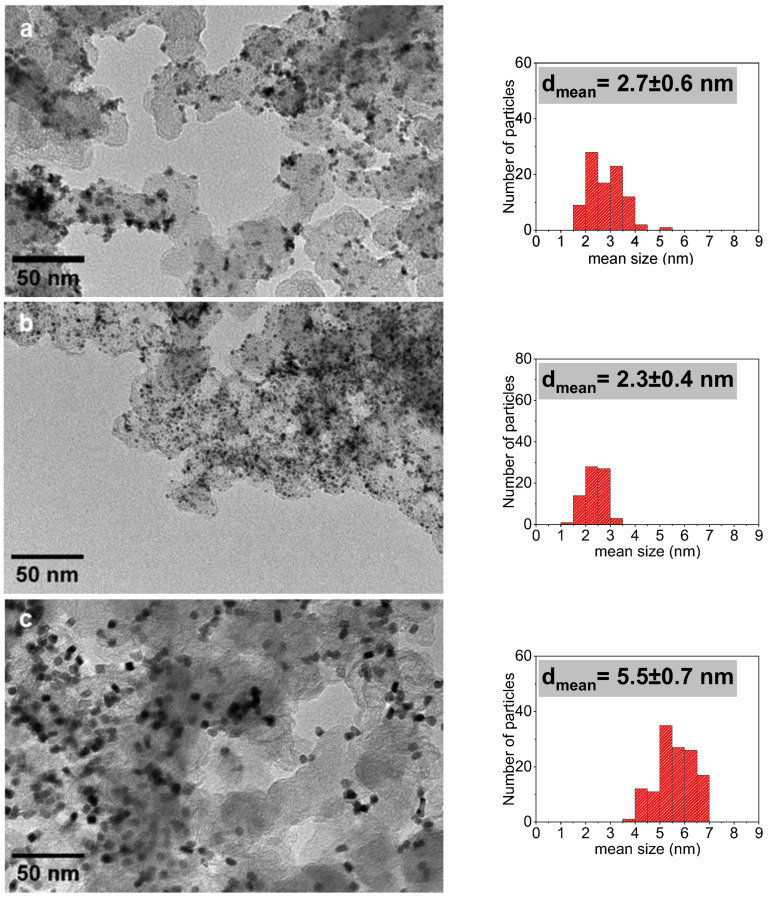
TEM images of Pt NPs formed by decomposition of [Pt(NBE)_3_] in toluene under 3 bar of H_2_ on KetjenBlack EC 300J: (**a**) by direct one-pot procedure without HDA, 29.6% Pt /KB; (**b**) by direct one-pot procedure with HDA, 28.6% Pt^HDA^/KB; (**c**) KB-supported Pt NPs prepared by an indirect two-step procedure (Pt/KB-I).

TEM analysis of the Pt^HDA^/KB-I nanomaterial obtained via the impregnation pathway (Figure 4c and Figure S4c; Table 1) showed well-dispersed NPs on the KB support with an average size of 5.5 ± 0.7 nm, close to that of the initial Pt NPs (4.9 ± 0.6 nm). Beside the size, the nanoparticles retained the cubic, faceted morphology of pristine Pt^HDA^ nanoparticles, in agreement with impregnation protocol. In contrast, KB-supported Pt nanoparticles prepared via the one-step pathway (Pt^HDA^/KB) yielded very well-dispersed Pt NPs with a smaller average size of 2.3 nm and more spherical shape (Figure 4b and Figure S4b; Table 1). This can be explained by the effect of the carbon support that may contribute to the dispersion of the Pt organometallic precursor and/or to the stabilization of the Pt NPs, thus limiting their growth to a lower size than in the case of HDA presence only. The KB-supported Pt nanoparticles prepared by the one-pot pathway but in the absence of HDA were found to be less-defined in shape and slightly larger in size, with a mean diameter of ca. 2.7 nm (Figure 4a and Figure S4a; Table 1) Given the difference in mean size observed for Pt/KB and Pt^HDA^/KB (2.7 nm vs. 2.3 nm; Figure 4; Table 1), one hypothesis could be that, if the KB support has influence on the formation of the NPs it does not have a primary role in the control of their growth, but rather in their dispersion. Interaction of HDA with the growing metal NPs is probably the key point for the limitation of the growth, while the carbon support may favor the dispersion of the precursor before its decomposition due to C-C interaction with the norbornene ligands. This may explain why, in the absence of HDA, poorly-defined Pt NPs are formed but not a bulk Pt material [25].

The XRD patterns of Pt^HDA^ and Pt^HDA^/KB-I nanomaterials (Figure 3) show a high degree of overlap, indicating that the crystal structure of Pt is well preserved upon deposition of the preformed HDA-stabilized Pt nanoparticles on the carbon support. The Scherrer equation method was also applied to determine the size of Pt crystallites in Pt^HDA^/KB-I, providing for 67.7° (2 2 0), 46.3° (2 0 0) and 39.9° (1 1 1), as 4.2 nm, 4.6 and 5.8 nm, respectively. These data also align with average particle size measured by TEM (5.5 nm), as previously observed for Pt^HDA^. Also, the diffractogram of KB-supported Pt nanoparticles synthesized in one-pot conditions without HDA (Pt/KB) shows an overlapping fcc structure compared to Pt^HDA^.

### 3.3. Electrochemical Characterization of Carbon-Supported Pt Nanoparticles

Electrochemical characterization in 0.1 M HClO_4_ was performed on KB-supported Pt nanomaterials synthesized via direct and indirect procedures, in comparison with a commercial reference presenting the same metal loading (30 wt.% Pt/C). The Pt electrochemical surface area (ECSA) was determined for three catalysts: Pt/KB and Pt^HDA^/KB, prepared by a one-pot synthesis procedure with and without HDA, respectively, and Pt^HDA^/KB-I, obtained via a two-step impregnation procedure (Table 2).

**Figure 5:**
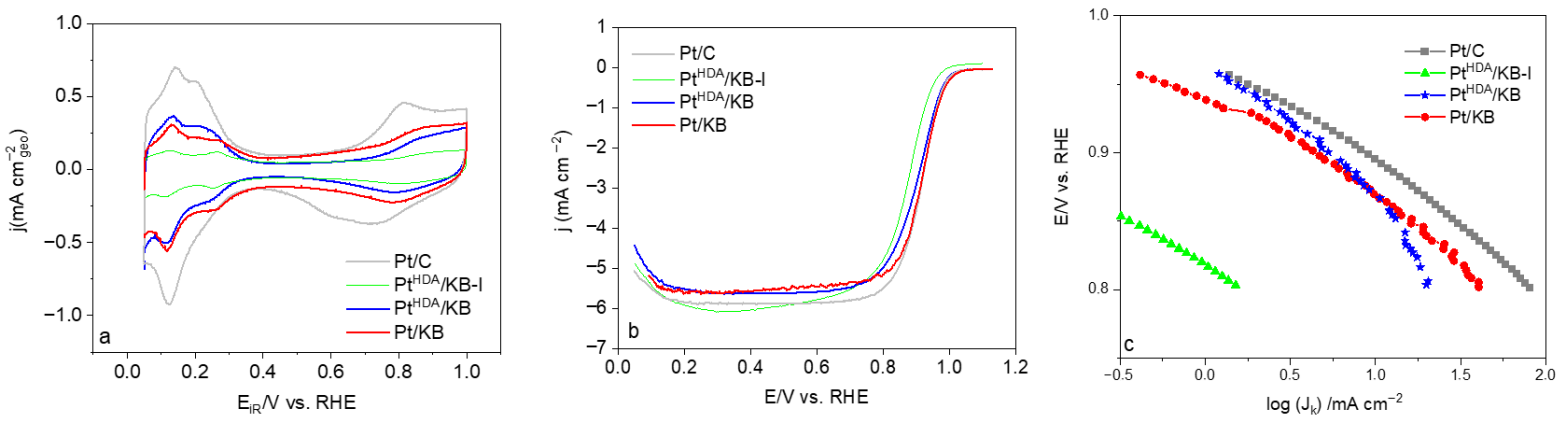
Electrochemical characterization of 30% Pt/C commercial reference, Pt^HDA^/KB-I, Pt^HDA^/KB and Pt/KB: (**a**) Cyclic voltammograms (CV) in N_2_ saturated 0.1 M HClO_4_ at 20 mV s^−1^, (**b**) ORR polarization curves in O_2_ saturated 0.1 M HClO_4_ at 20 mV s^−1^ and 1600 rpm; (**c**) mass transport corrected ORR Tafel plots.

The calculated average ECSA values were 50, 88, and 20 m^2^g_pt_^−1^ for Pt/KB, Pt^HDA^/KB, and Pt^HDA^/KB-I, respectively (Table 2, Figure 5). All the values are lower than that determined for the Pt/C reference (96 ± 8), which can be ascribed to the lower size of the Pt nanoparticles in this reference catalyst (d_mean_ = 1.7 ± 0.4 nm, Figure S2).

Among the synthesized nanomaterials, Pt^HDA^/KB exhibits the highest ECSA of 88 m^2^·gPₜ^−1^; a value that correlates with the smaller nanoparticle size in this nanomaterial in comparison to Pt/KB and Pt^HDA^/KB-I counterparts (see Figure 4). Theoretically, the ECSA for Pt^HDA^/KB—assuming a completely clean and accessible surface—is calculated to be 121 m^2^ g_pt_^−1^ (based on the average diameter of 2.3 nm and assuming a spherical morphology, Equation (8)), while Pt^HDA^/KB-I, with a cubic morphology and an edge length of 5.5 nm, yields a theoretical ECSA of 51 m^2^ g_pt_^−1^ (Equation (9)). The observed ECSA trend aligns with both particle size and literature findings, as smaller platinum nanoparticles inherently provide greater specific surface areas [30]. The fact that the calculated ECSA is close to the theoretical maximum suggests that hexadecylamine does not completely block the active sites, which is noteworthy. In contrast, the Pt/KB sample synthesized without HDA displays a significantly lower ECSA of 50 m^2^ g_pt_^−1^ (vs. the theoretical 103 m^2^ g_pt_^−1^ with the spherical shape approximation), which may be attributed to particle aggregation in the absence of stabilizer (see Figure 4a). These results underscore the dual role of HDA in preventing particle agglomeration and promoting the exposure of active surface sites.

In addition, ORR electrochemical activity was evaluated using rotating disk electrode (RDE) measurements in 0.1 M HClO_4_ (Figure 5, Table 2) [31]. The Tafel slopes for Pt/C and Pt/KB in the low-current region were approximately 60 mV dec^–1^ (Table 2), in agreement with already reported results on supported platinum particles [32]. However, for Pt^HDA^/KB the value is quite high (91 mV dec^−1^), which can be related to the presence of the HDA at the Pt surface modifying surface chemistry and adsorption of intermediates, thus shifting apparent kinetics away from the ideal ~60 mV dec^−1^ behavior [33].

For a more quantitative discussion, the ORR activities of the different catalysts were determined by measuring the current density at 0.9 V/RHE (Table 2). The specific activity I_s_ values were found to be very similar in average for commercial Pt/C and Pt^HDA^/KB, around 0.4 mA cm^−2^_Pt_. The catalyst prepared in the absence of HDA (Pt/KB) showed a slightly higher value of 0.5 mA cm^−2^, and that prepared by impregnation (Pt^HDA^/KB-I) presented the highest (0.7 mA cm^−2^), in agreement with the fact that larger nanoparticles have less defects and higher I_s_. The ORR mass activity, I_m_, follows a different trend. The I_m_ was found to be slightly lower for Pt/KB (0.25 A mg^−1^_Pt_) than for Pt^HDA^/KB (0.35 A mg^−1^_Pt_), meaning that the catalyst synthetized using HDA presents higher intrinsic catalytic activity per unit of mass [34]. Such activity is slightly higher than that of the commercial Pt/C (0.34 A mg^−1^_Pt_). The impregnated nanomaterial (Pt^HDA^/KB-I) displays a very low I_m_ (0.1 A mg^−1^_Pt_), which is consistent with its larger-sized NPs and the exposed crystal planes (5 nm-sized cubes). Another metric that can be used to evaluate the different Pt catalysts is the ORR half-wave potential (E_1_/_2_), reflecting the intrinsic ORR activity (kinetics-dominated region). The E_1_/_2_ values are similar for all the prepared nanomaterials and in agreement with reported data on supported Pt catalysts (0.90 V/RHE) [35], except for Pt^HDA^/KB-I which displays the lowest ORR activity (E_1_/_2_ = 0.87 V/RHE) as already discussed.

The differences in electroactivity for nanomaterials with very close nanoparticle sizes, Pt/KB and Pt^HDA^/KB indicate that: (1) Particle size alone does not affect electrochemical activity, and (2) the combination of support and synthesis conditions (e.g., use of stabilizer) plays a key role in the ORR performance [35]. The nature of the exposed crystal facets, which influence the catalytic performance, is closely related to particle size [36].

The particle size effect on Pt ORR activity has been widely reported, though the optimal size range remains debated [37]. Several studies indicate that ORR activity decreases for Pt NPs larger than 4 nm because of their reduced surface-to-volume ratio and changes in the surface structure affecting specific activity [38]. DFT studies [39] show that smaller particles expose more under-coordinated sites (steps, edges, kinks), which bind HO* intermediates of ORR too strongly [30], increasing the overpotential for its removal. As a result, a decrease in particle size should induce a decrease in specific activity, while mass activity peaks at ~3 nm. This aligns with experimental findings [40] and with a favorable distribution of (1 1 1) and (1 0 0) facets in cuboctahedral particles [41]. However, this does not apply to cubic nanoparticles, with the dominating (1 0 0) facet being the least active in HClO_4_ [42].

**Figure 6:**
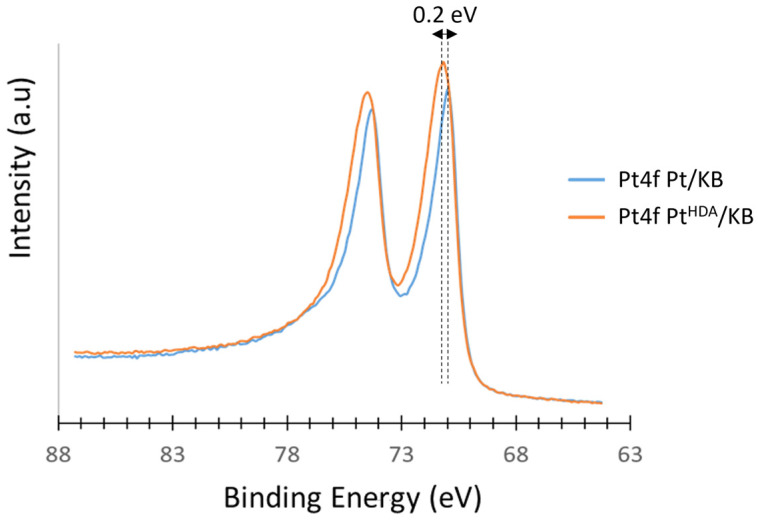
Comparison of Pt4f XPS spectra of as synthesized Pt/KB (blue curve) and Pt^HDA^/KB (orange curve) nanomaterials.

Additionally, HDA, as an organic ligand, can modify the electronic structure of Pt NPs through charge transfer or ligand-metal interactions. This may shift the d-band center of platinum, influencing adsorbate binding energies and catalytic performance. Otherwise, residual HDA may partially block active sites, reducing accessibility for reactants and altering the apparent electronic properties of the Pt surface. The ECSA results indicate that most surface sites remain accessible, possibly due to the removal of excess HDA or its lability at the Pt surface [25]. In order to assess the effect of the presence of residual HDA on the electrocatalytic properties of Pt^HDA^/KB, X-ray photoelectron spectroscopy (XPS) analysis was performed on the catalyst in comparison to its HDA-free counterpart, Pt/KB. XPS spectra and elemental quantification data are provided in the Supplementary Materials (see Section S6). Figure 6 represents a superposition of the Pt4f region of the XPS spectra of Pt/KB (blue curve) and Pt^HDA^/KB (orange curve). The observed Pt4f peaks in both cases are attributed to the supported Pt nanoparticles. Figure 6 shows a shift of +0.2 eV for the Pt4f signal of Pt^HDA^/KB (B.E. 71.2 eV) compared to the Pt4f signal of Pt/KB (B.E: 71 eV). The shift observed in the presence of the amine may be attributed to the electron transfer from Pt to the carbon support, which is enhanced by the presence of more electronegative nitrogen atoms from HDA. Such an effect has been already reported for Pt nanoparticles deposited on nitrogen-functionalized supports [43,44], affecting their ORR activity and stability. This may explain the slightly higher electrocatalytic activity of Pt^HDA^/KB compared to the otherwise identical catalyst synthetized in the absence of the amine.

**Figure 7:**
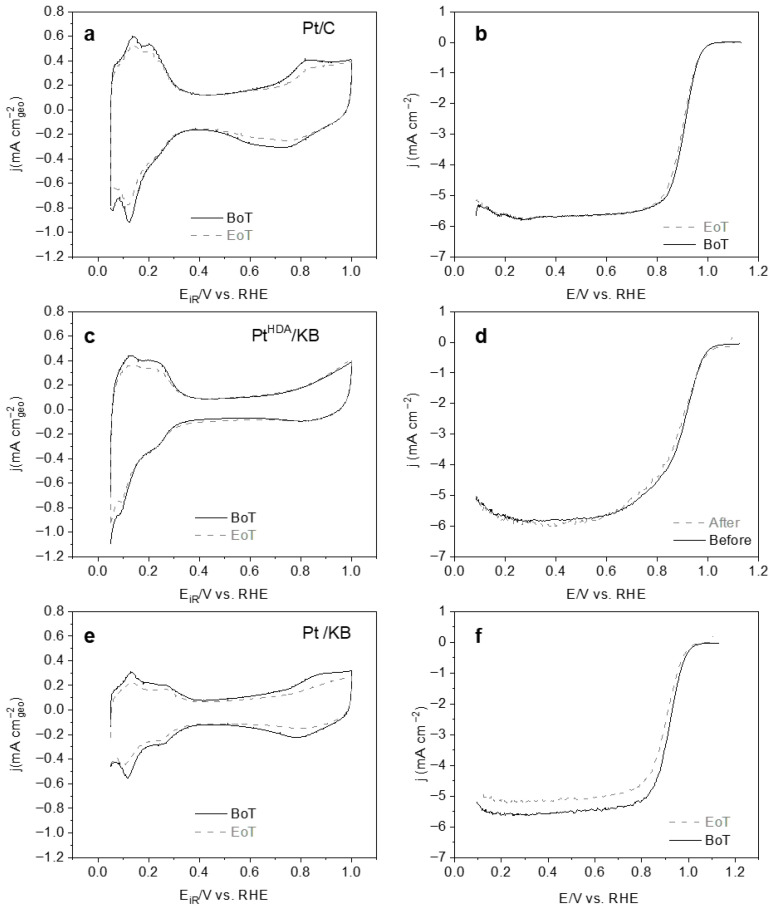
CVs and ORR polarization curves before (BoT) and after AST tests (EoT) of 10,000 cycles (0.6 and 0.925 V/RHE at 100 mV·s^−1^ scan rate and 1600 rpm) in 1 M HClO_4_ for 30% Pt/C (**a**,**b**), Pt^HDA^/KB (**c**,**d**), and Pt /KB (**e**,**f**).

Alongside electroactivity, durability is a relevant factor for a potential PEMFC catalyst. In this work, it was evaluated for the two most active nanocatalysts, Pt^HDA^/KB and Pt/KB. In order to assess the electrocatalyst durability with time, accelerated stress tests (AST) consisting of prolonged potential cycling are commonly performed [9]. Depending on the specific conditions (temperature, voltage limits, atmosphere) they accelerate key degradation mechanisms, such as Pt nanoparticle dissolution, reprecipitation, agglomeration, or support corrosion, which leads to a loss of electrochemically active surface area and overall electroactivity [45]. The Pt electrochemical stability to dissolution was evaluated for Pt^HDA^/KB and Pt/KB catalysts using an AST consisting of 10,000 voltage cycles between 0.6 and 0.925 V/RHE at 100 mV·s^−1^ scan rate in Ar-saturated 0.1 M HClO_4_. ECSA and ORR activity evolution [46] was monitored. This potential window was selected to focus on Pt degradation processes, while surpassing 1.0 V/RHE drives carbon support corrosion. CVs and ORR polarization curves were recorded before (beginning of test, BoT) and after AST testing (end of test, EoT) (Figure 7, Table 2) to assess the evolution in ECSA and electrocatalytic activity upon cycling.

The synthesized KB-supported Pt nanomaterials lost more electrochemical surface area (−18% for Pt^HDA^/KB and −20% for Pt/KB) than the Pt/C material (−13%) (Table 2), but retained more specific activity (5% vs. 12% loss for the reference catalyst). For all the nanomaterials investigated, the mass activity drop at EoT was around 23%. The ORR half-wave potential slightly decreased for all catalysts, and at a higher extent for the Pt/KB catalyst (from 0.9 to 0.87 V/RHE).

Although the Pt^HDA^/KB and Pt/KB catalysts showed a larger loss in ECSA compared to the commercial Pt/C material, their mass activity remained quite stable after AST cycling, with Pt^HDA^/KB displaying the highest EoT value (0.27 A mg_Pt_^−1^ at 0.9 V/RHE). The small decrease in specific activity indicates that their surface activity is quite well preserved. Overall, both nanoparticle size distribution and surface stabilization played a relevant role in the long-term stability of the electrocatalysts that were developed.

All in all, these results suggest that the addition of HDA for the synthesis of KB-supported Pt nanoparticles has a beneficial effect on their catalytic behavior in ORR, both in terms of activity (Pt^HDA^/KB having a higher mass activity than Pt/KB) as well as stability, as shown through accelerated stress tests (higher ECSA retention and EoT mass activity for Pt^HDA^/KB than Pt/KB).

## 4. Conclusions

This work confirms the efficiency of the organometallic approach for the synthesis of Pt-based nanomaterials with potential use in ORR electrocatalysis.

Two different strategies were followed for the synthesis of carbon (KetjenBlack EC-300J KB)-supported Pt nanocatalysts using the [Pt(NBE)_3_] organometallic complex as the Pt atom source, which were released when treating this complex under hydrogen atmosphere. On one hand, a one-step synthesis was performed in the presence of the support with (Pt^HDA^/KB) or without (Pt/KB) the HDA stabilizer. On the other hand, a two-step synthesis based on the impregnation of KB with a colloidal suspension of preformed Pt^HDA^ (Pt^HDA^/KB-I) was carried out. In all cases, Pt nanoparticles were very well dispersed on the carbon surface. The one-pot route presents several advantages including simplicity, reduced number of synthesis steps, fast kinetics, and scalability. In addition, the direct growth of Pt nanoparticles in the presence of the carbon support promotes Pt–C bonds, which is beneficial for efficient electron transfer during electrocatalysis.

The ORR electroactivity of all synthesized nanomaterials was evaluated by rotating disk electrode (RDE). The Pt nanomaterial prepared by the two-step/impregnation method (Pt^HDA^/KB-I) showed a lower ORR activity than the ones prepared by the direct synthesis method (Pt^HDA^/KB and Pt/KB). The Pt^HDA^/KB nanomaterial showed a slightly better dispersion of the Pt nanoparticles on the KB, associated with a higher electrochemical performance and ECSA values of 86 m^2^_pt_g_pt_^−1^, while the stabilizer-free nanomaterial (Pt/KB) reached an ECSA value of 50 m^2^_pt_g_pt_^−1^. The optimized ORR properties of the Pt^HDA^/KB versus the Pt/KB are attributed to the influence of the HDA ligand added at the synthesis step, which has a beneficial effect on the electronic structure of the Pt sites and also improves the stability under turnover conditions.

Furthermore, Pt/KB and Pt^HDA^/KB were subjected to an accelerated stress test, demonstrating similar activity and durability than the commercial 30% Pt/C benchmark, with slightly higher specific activity retention.

Overall, this study validates the organometallic synthesis approach, using stabilizing ligands to achieve control over metal nanoparticles features and improve catalytic properties, as a promising direction for achieving Pt-based nanocatalysts of significant attractiveness for electrocatalytic applications, as proven by evaluation in ORR.

## Figures and Tables

**Table 1:** Mean size of Pt NPs and Pt content as determined by ICP-AES in KB-supported Pt nanomaterials.

Pt Nanomaterial	Pt NP Mean Size nm	ICP-AES wt.%
Pt^HDA^/KB	2.3 ± 0.4	28.6
Pt/KB	2.7 ± 0.6	29.6
Pt^HDA^/KB-I	5.5 ± 0.7	28.8
Pt^HDA^	4.9 ± 0.6	84.0

**Table 2:** ECSA and ORR kinetic parameters of 30 wt% Pt nanocatalysts at BoT (and EoT).

Nanocatalyst	ECSA (m^2^g_pt_^−1^)	Tafel Slope (mV dec^–1^)	I_s_ (mA cm^−2^_Pt_) @ 0.9 V/RHE	I_m_ (A mg_Pt_^−1^) @ 0.9 V/RHE	E_1_/_2_ (V/RHE)
Pt^HDA^/KB-I	20 ± 1	-	0.70 ± 0.01 (-)	0.10 ± 0.01 (-)	0.87
Pt^HDA^/KB	88 ± 2 (72)	91	0.39 ± 0.05 (0.37)	0.35 ± 0.03 (0.27)	0.90 ± 0.007 (0.89)
Pt/KB	50 ± 1 (40)	57	0.49 ± 0.05 (0.46)	0.25 ± 0.03 (0.19)	0.90 ± 0.006 (0.87)
Pt/C	96 ± 8 (84)	67	0.34 ± 0.03 (0.30)	0.34 ± 0.03 (0.26)	0.91 ± 0.005 (0.90)

Note: Values preceded by ± are standard deviations.

## Data Availability

Data available on request.

## Supplementary Materials

Supporting information can be downloaded at: https://www.mdpi.com/article/10.3390/nano16060364/s1. Figure S1. Atomic force microscopy (AFM) studies of single Pt^HDA^ nanoparticles. Figure S2. TEM image and size distribution histogram of commercial 30% wt. Pt/C. Figure S3. From top to bottom, FT-IR spectra of Pt^HDA^ NPs (purple) in comparison to those of resulting supernatant after washings of Pt^HDA^ NPs (grey), pure HDA used as stabilizer (blue) and [Pt(NBE)_3_] complex used as Pt precursor (black). Figure S4. (a). TEM image of 30 wt.%Pt/KB. (b). TEM image of 30 wt.%Pt^HDA^/KB. (c). TEM image of 30 wt.%Pt^HDA^/KB-I. Figure S5. Electrochemical characterization of all Pt nanocatalysts: (a) Cyclic voltammograms (CV) in N_2_—saturated 0.1 M HClO_4_ at 20 mV s^−1^, (b) ORR polarization curves in O_2_—saturated 0.1 M HClO_4_ at 20 mV s^−1^ at 1600 rpm. Table S1. ECSA and ORR kinetic parameters of carbon-supported Pt nanocatalysts prepared with KB and in the presence of HDA by direct synthesis method (Pt^HDA^/KB nanocatalysts series in a range of 30 wt.%Pt to 60 wt.%Pt, as determined by ICP-AES analysis). Figure S6. (a). XPS survey spectrum of 30 wt.%Pt/KB nanomaterial. (b). Pt4f spectrum of 30 wt.%Pt/KB nanomaterial. Table S2. (a). XPS elemental quantification of 30 wt.%Pt/KB nanomaterial. (b). XPS elemental quantification of 30 wt.%Pt^HDA^/KB nanomaterial. Figure S7. (a). XPS survey spectrum of 30 wt.%Pt^HDA^/KB nanomaterial. (b). Pt4f spectrum of 30 wt.%Pt^HDA^/KB nanomaterial. Table S3. Figures of merit of Pt/C catalysts, as extracted from a recent article. Refs. [47,48] are cited in the supplementary materials.

## Abbreviations

The following abbreviations are used in this manuscript:

[Pt(NBE)_3_]	tris(norbornene)platinum
NPs	Nanoparticles
HDA	Hexadecylamine
TEM	Transmission Electron Microscopy
HR-TEM	High-Resolution Transmission Electron Microscopy
ICP-AES	Inductively Coupled Plasma-Atomic Emission Spectroscopy
RDE	Rotating Disk Electrode
RHE	Reversible Hydrogen Electrode
S_bet_	Specific surface area
BET	Brunauer–Emmett–Teller
ORR	Oxygen reduction reaction
PEMFC	Proton exchange membrane fuel cell
