# Supplementary material for: Organometallic Synthesis of Platinum-Based Nanomaterials for the Oxygen Reduction Reaction
Source: Nanomaterials (Basel). 2026 Mar 17;16(6):364. doi: 10.3390/nano16060364 (PMC13029607; doi:10.3390/nano16060364)
Supplement: Supplementary file 1 [file nanomaterials-16-00364-s001.zip › nanomaterials-4151434-supplementary.pdf]

Supplementary Materials

Organometallic synthesis of platinum-based nanomaterials for the oxygen reduction reaction

Nargiz Kazimova[1,2], Nuria Romero[1], Jérôme Esvan[3], Marjorie Cavarroc[4], Sara Cavaliere[2,*], Karine Philippot[1,*]

[1] CNRS, LCC (Laboratoire de Chimie de Coordination), UPR8241, University of Toulouse, UPS, INPT, CEDEX 4, 31077 Toulouse, France
[2] ICGM, University Montpellier, CNRS, ENSCM, CEDEX 5, 34095 Montpellier, France
[3] CIRIMAT, Université de Toulouse, CNRS-INPT-UPS, 4 Allée Emile Monso, BP 44362 31030 Toulouse, France
[4] Safran-Tech, Rue des jeunes bois, 78772, Magny les Hameaux, France

*sara.cavaliere@umontpellier.fr; karine.philippot@lcc-toulouse.fr

Table of contents

S1. Atomic force microscopy (AFM) studies of Pt^{HDA} nanoparticles 2
S2. TEM study of 30 wt.%Pt/C commercial reference 3
S3. Fourier-transform infrared spectroscopy (FT-IR) 4
S4. Low magnification TEM images of 30 wt.%Pt/KB, 30 wt.%Pt^{HDA}/KB and 30 wt.%Pt^{HDA}/KB-I 6
S5. Electrochemical studies 8
S6. XPS studies of 30 wt.%Pt/KB and 30 wt.%Pt^{HDA}/KB nanomaterials 10
S7. Comparison of figures of merit of recently reported Pt catalysts in ORR 13
S8. References 13

S1. Atomic force microscopy (AFM) studies of Pt^{HDA} nanoparticles

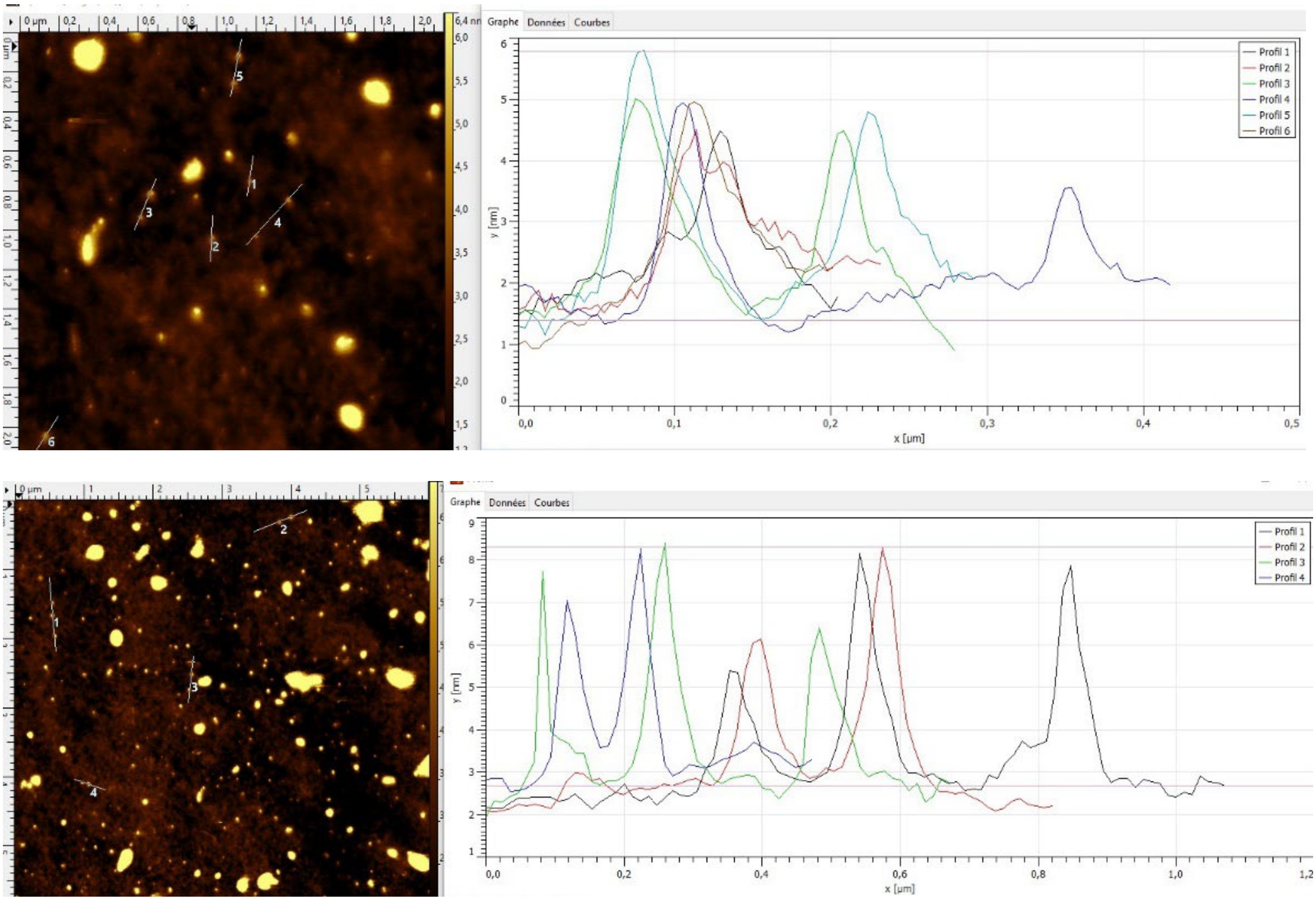

Figure S1. Atomic force microscopy (AFM) studies of single Pt^{HDA} nanoparticles.

S2. TEM study of 30 wt.%Pt/C commercial reference

TEM analysis of 30% Pt/C (from Johnson Matthey) showed the presence of well dispersed Pt nanoparticles of 1.7 ± 0.4 nm on the carbon surface.

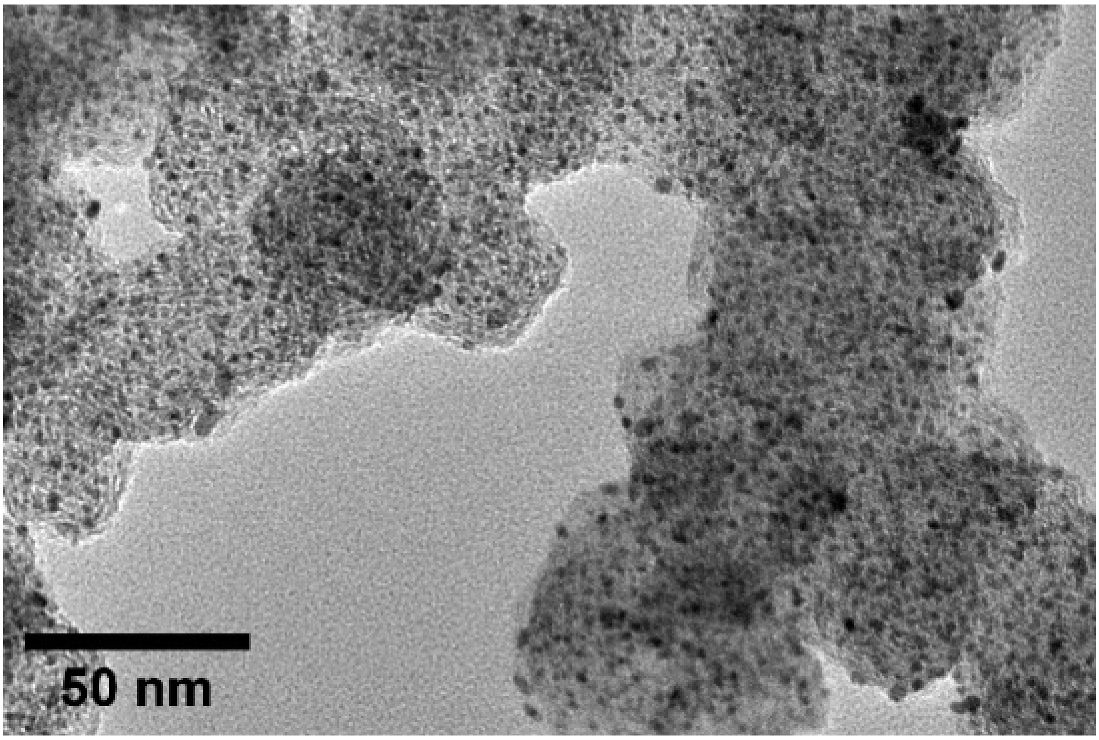

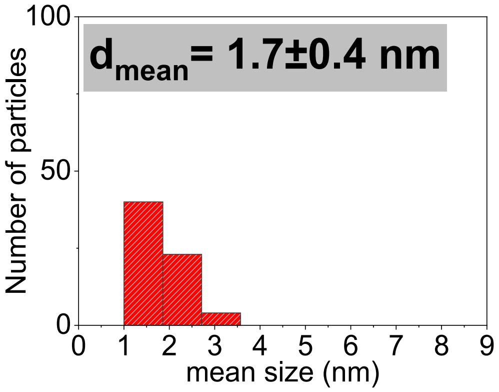

Figure S2. TEM image and size distribution histogram of commercial 30% wt. Pt/C.

S3. Fourier-transform infrared spectroscopy (FT-IR)

Fourier-transform infrared spectroscopy (FT-IR) spectra were recorded in ATR mode for both the isolated Pt^{HDA} NPs in a solid state and the vacuum dried supernatants of the reaction, and then compared to those of pure $[Pt(NBE)_3]$ complex and pure HDA stabilizer (Figure S3). The spectrum of the Pt^{HDA} NPs (in purple) is very flat, thus indicating a low amount of organic compounds at the Pt surface. In contrast, the spectrum of the reaction supernatants resulting from the washings of Pt^{HDA} NPs with pentane (in grey) clearly shows the presence of peaks which can be attributed to HDA (see blue spectrum). These FT-IR results indicate that only a low quantity of HDA stabilizes the Pt^{HDA} NP surface and that the washings performed for the purification of the Pt^{HDA} NPs allowed to remove the excess of HDA.

Attribution of vibration bands of pure HDA (blue spectrum) : the peaks between 3100 and 3300 cm^{-1} correspond to $N(-H)_2$ antisymmetric and symmetric stretching, the sharp peak at 1462 cm^{-1} is attributed to $N(-H)_2$ scissoring, the peaks at 923 cm^{-1} to $N(-H)_2$ wagging,[1] the peak at 2953 cm^{-1} is ascribed to $C(-H)_3$ stretching, the peak at 1058 cm^{-1} corresponds to C-N stretching and those at 718 cm^{-1} correspond to $C(-H)_2$ rocking in the carbon chain.

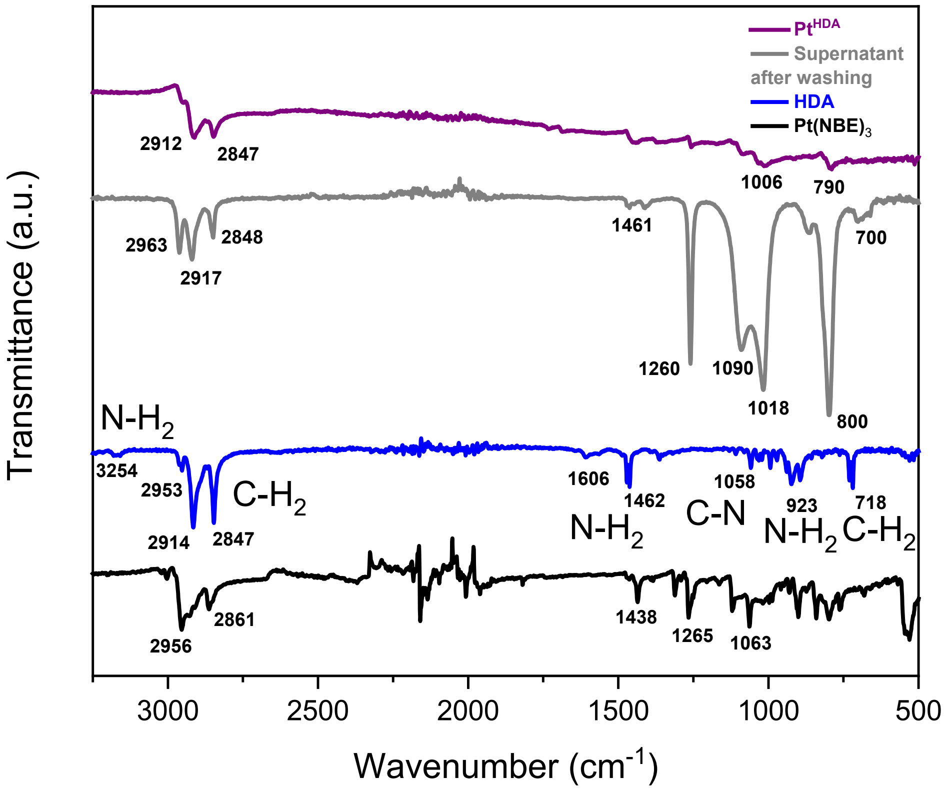

Figure S3. From top to bottom, FT-IR spectra of Pt^{HDA} NPs (purple) in comparison to those of resulting supernatant after washings of Pt^{HDA} NPs (grey), pure HDA used as stabilizer (blue) and [Pt(NBE)$_3$] complex used as Pt precursor (black).

S4. Low magnification TEM images of 30 wt.%Pt/KB, 30 wt.%Pt^{HDA}/KB and 30 wt.%Pt^{HDA}/KB-I

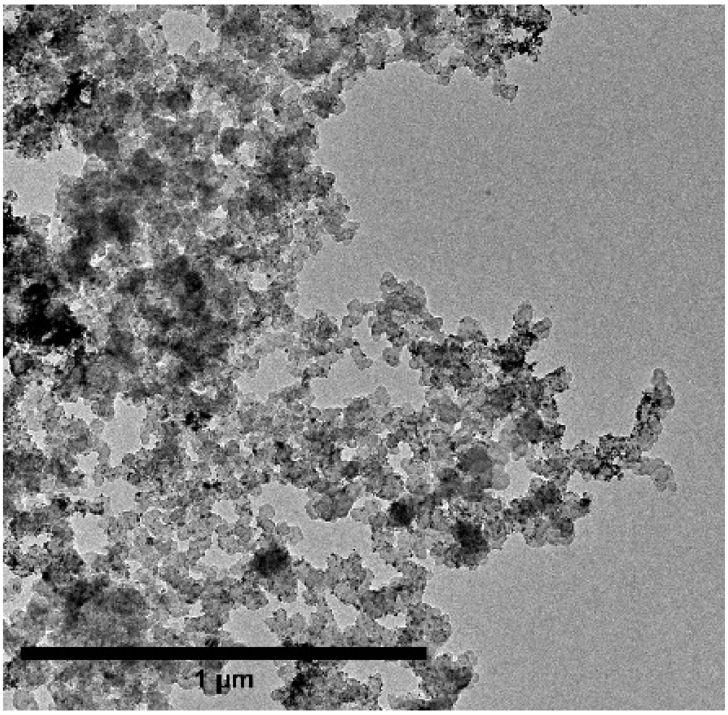

Figure S4a. TEM image of 30 wt.%Pt/KB

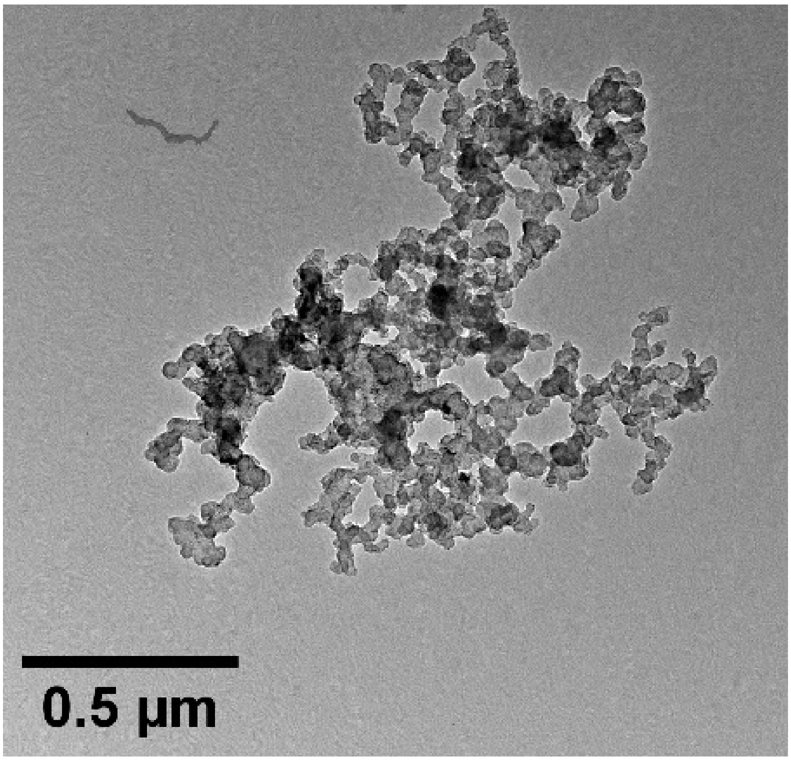

Figure S4b. TEM image of 30 wt.%Pt^{HDA}/KB

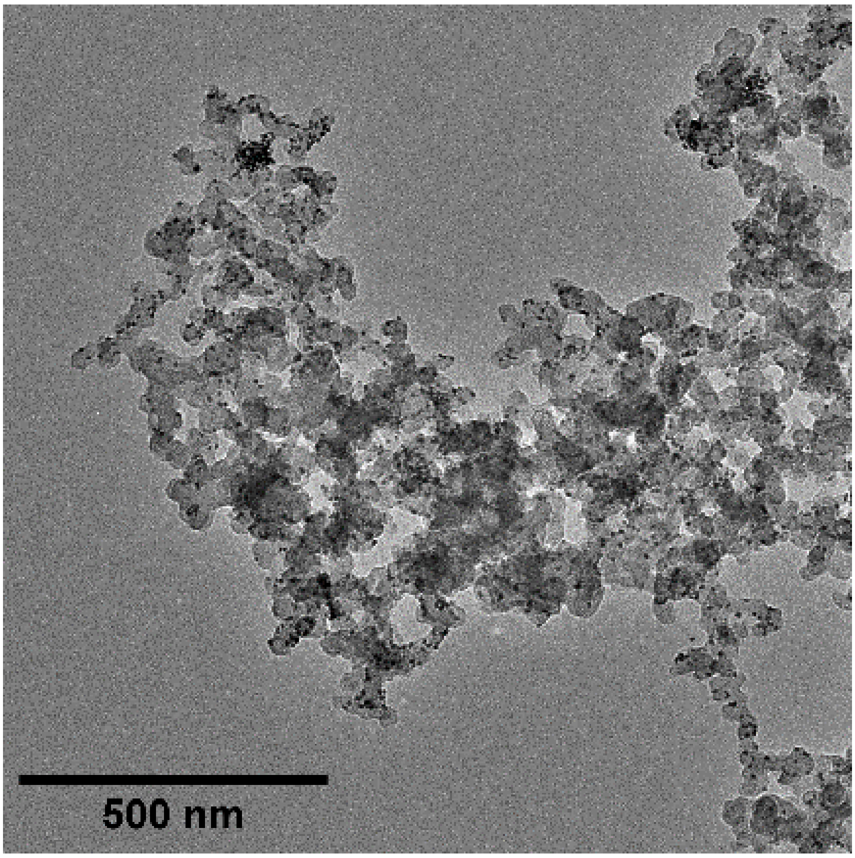

Figure S4c. TEM image of 30 wt.%Pt^{HDA}/KB-I

S5. Electrochemical studies

Figure S5 presents the electrochemical characterization of the Pt^{HDA}/KB nanomaterials with different Pt loadings (30 wt.% to 60 wt.%), for comparison purpose. The results show that the nanomaterial prepared with 30 wt.% Pt is the most promising as ORR catalyst.

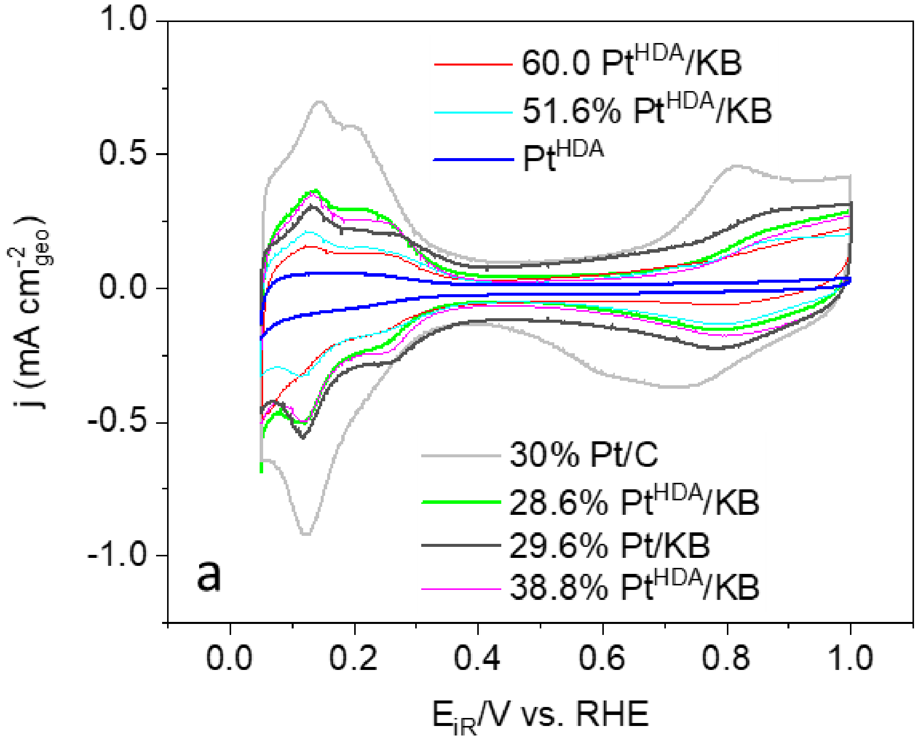

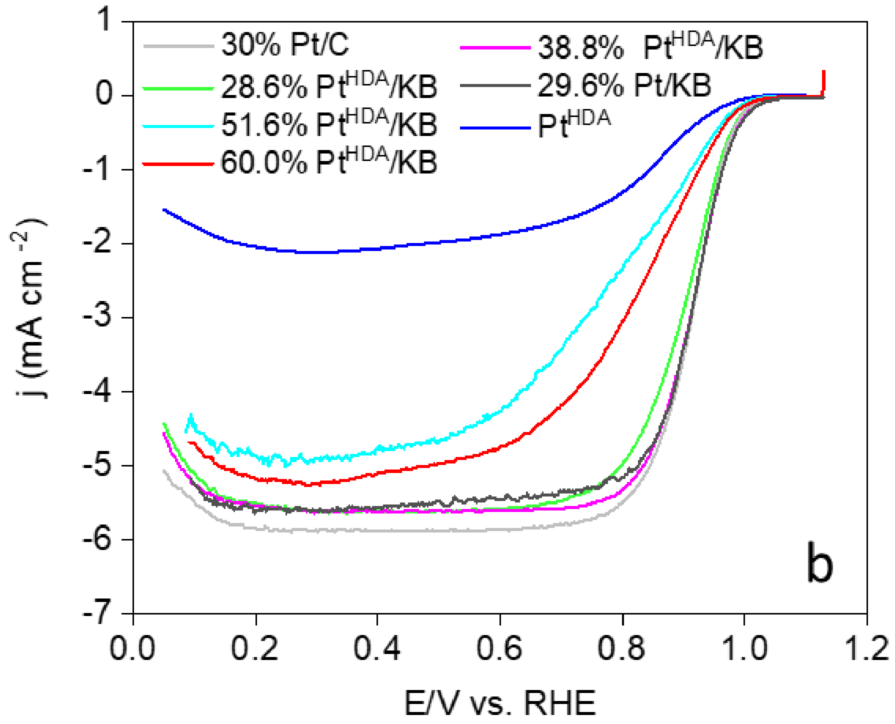

Figure S5. Electrochemical characterization of all Pt nanocatalysts: a) Cyclic voltammograms (CV) in N_2 - saturated 0.1 M $HClO_4$ at 20 mV s^{-1}, b) ORR polarization curves in O_2 - saturated 0.1 M $HClO_4$ at 20 mV s^{-1} at 1600 rpm.

Table S1. ECSA and ORR kinetic parameters of carbon-supported Pt nanocatalysts prepared with KB and in the presence of HDA by direct synthesis method (**Pt^{HDA}/KB** nanocatalysts series in a range of 30 wt.%Pt to 60 wt.%Pt, as determined by ICP-AES analysis).

	**Pt loading %wt.**	**ECSA** $m^2_{pt}g_{pt}^{-1}$	I_s (mA cm^{-2}_{Pt}) @ 0.9 V/RHE	I_m (A/mg_{Pt}) @ 0.9 V/RHE
**Pt^{HDA}/KB nanocatalysts**	30	112	0.4	0.4
	38.8	82	0.5	0.4
	51.6	31	0.2	0.05
	60.0	18	0.2	0.04

S6. XPS studies of 30 wt.%Pt/KB and 30 wt.%Pt^{HDA}/KB nanomaterials

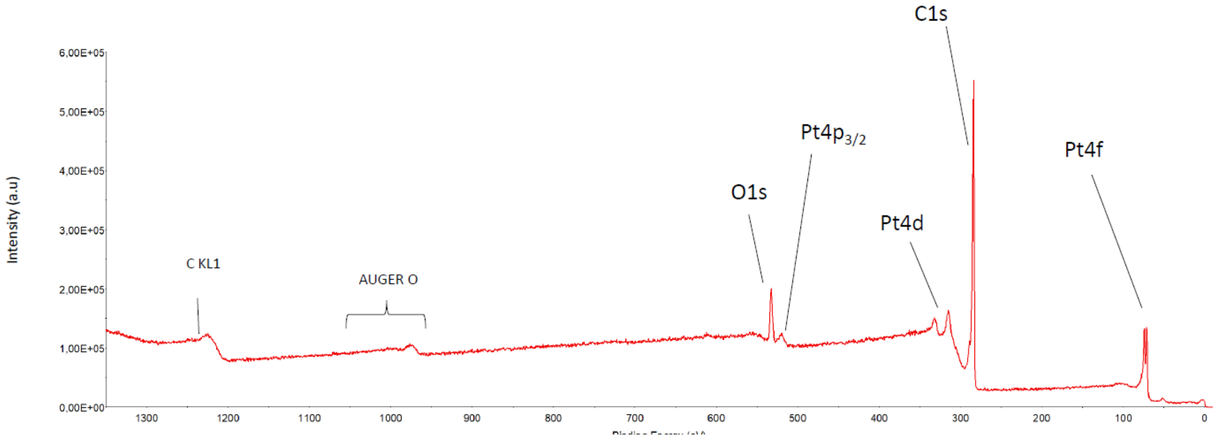

Figure S6a. XPS survey spectrum of 30 wt.%Pt/KB nanomaterial

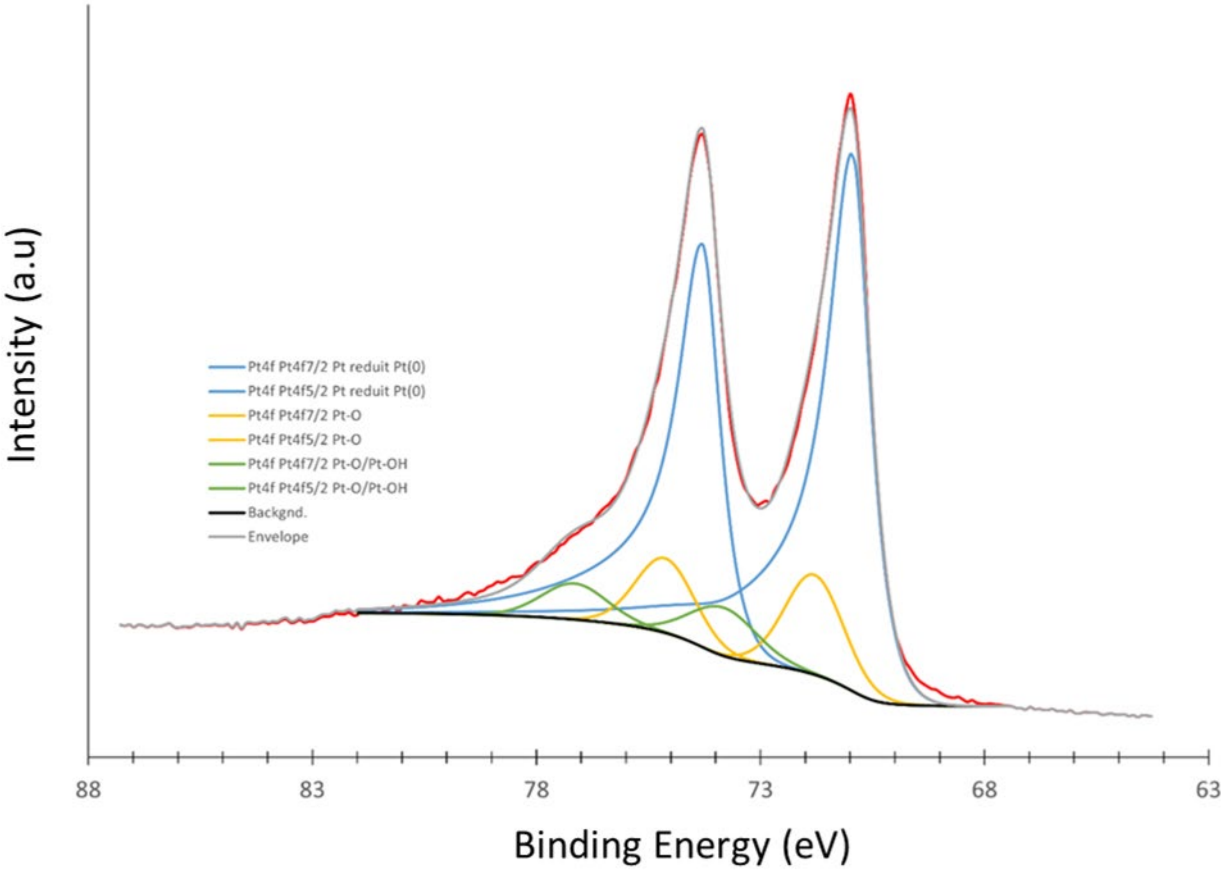

Figure S6b. Pt4f spectrum of 30 wt.%Pt/KB nanomaterial

Table S2a. XPS elemental quantification of 30 wt.%Pt/KB nanomaterial

Peak	Peak BE (eV)	FWHM (eV)	Atomic %
Pt4f$_{7/2}$ Pt(0)	71,0	1,2	1,0
Pt4f$_{7/2}$ Pt-O	71,8	1,5	0,2
Pt4f$_{7/2}$ Pt-O/Pt-OH	73,8	1,7	0,1
Pt4f$_{5/2}$ Pt(0)	74,3	1,2	0,7
Pt4f$_{5/2}$ Pt-O	75,1	1,5	0,1
Pt4f$_{5/2}$ Pt-O/Pt-OH	77,2	1,7	0,1
C1s	284,2	1,0	88,9
O1s	532,8	3,4	9,0

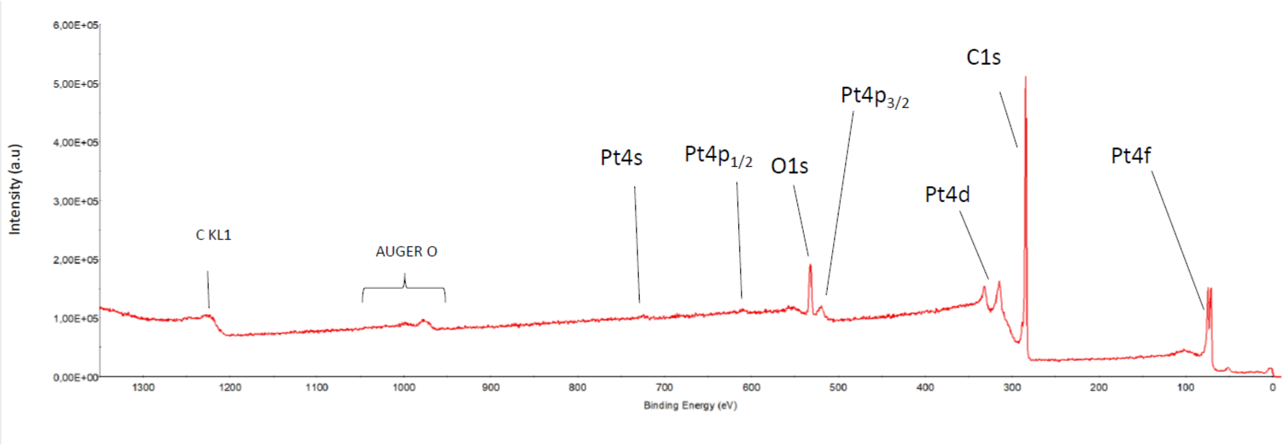

Figure S7a. XPS survey spectrum of 30 wt.%Pt^{HDA}/KB nanomaterial

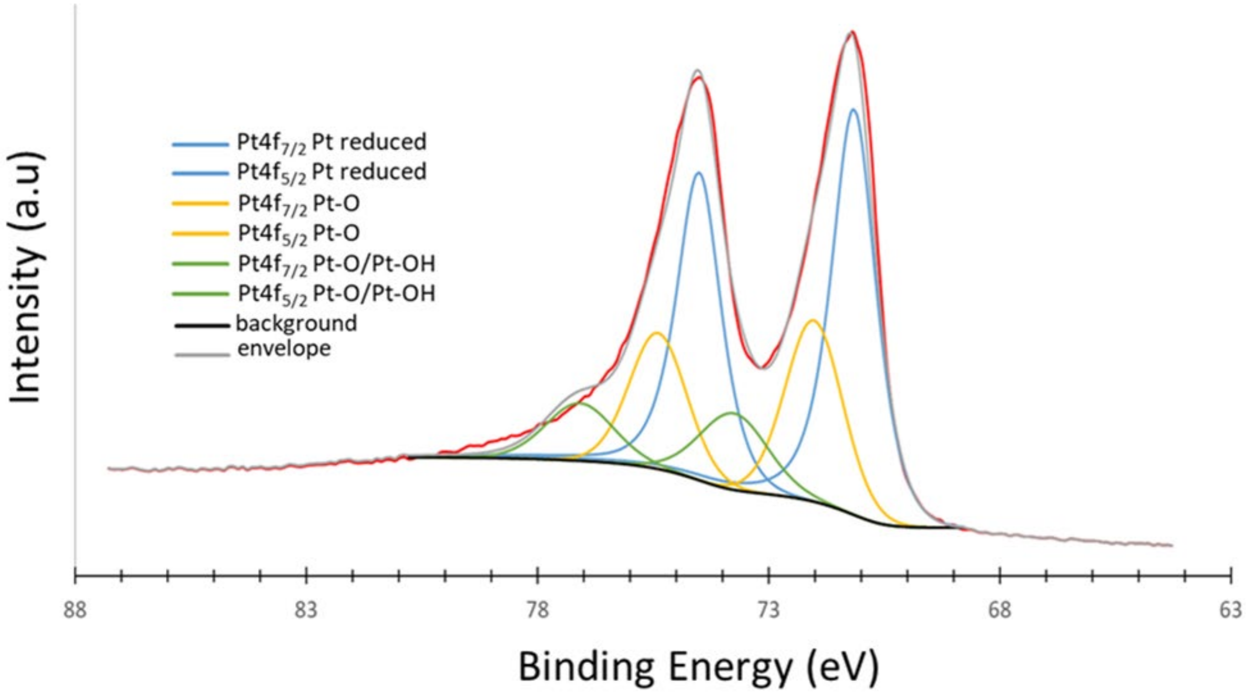

Figure S7b. Pt4f spectrum of 30 wt.%Pt^{HDA}/KB nanomaterial

Table S2b. XPS elemental quantification of 30 wt.%Pt^{HDA}/KB nanomaterial

Peak	Peak BE (eV)	FWHM (eV)	Atomic %
$Pt4f_{7/2}$ Pt(0)	71,0	1,2	1,2
$Pt4f_{7/2}$ Pt-O	71,8	1,4	0,4
$Pt4f_{7/2}$ Pt-O/Pt-OH	73,8	1,5	0,1
$Pt4f_{5/2}$ Pt(0)	74,4	1,2	0,9
$Pt4f_{5/2}$ Pt-O	75,1	1,4	0,3
$Pt4f_{5/2}$ Pt-O/Pt-OH	77,1	1,5	0,1
C1s	284,2	1,2	86,7
N1s	398,1	1,3	0,6
O1s	532,4	3,3	9,9

S7. Comparison of figures of merit of recently reported Pt catalysts in ORR

Pt/C Catalyst	Manufacturer	L_{Pt} [μg_{Pt} cm^{-2}]	ECA [m^2 g_{Pt}^{-1}]	I_s (mA $cm^{-2}{}_{Pt}$) at 0.9 V/RHE	I_m (A mg_{Pt}^{-1}) at 0.9 V/RHE
46.4 wt% Pt/HSC	Tanaka Kikinzoku Kogyo (TKK)	18	99 ± 5	0.485 ± 0.050	0.477 ± 0.042
37.8 wt% Pt/HSC	Johnson Matthey (JM)	18	125 ± 3	0.515 ± 0.026	0.646 ± 0.035
47.2 wt% Pt/HSC	Umicore	18	59 ± 2	0.344 ± 0.028	0.202 ± 0.021

Table S3. Figures of merit of Pt/C catalysts, as extracted from a recent article.[2]

S8. References

(1) Eck, M. Performance Enhancement of Hybrid Nanocrystal-Polymer Bulk Heterojunction Solar Cells : Aspects of Device Efficiency, Reproducibility, and Stability, 2014.

(2) Kocha, S. S.; Shinozaki, K.; Zack, J. W.; Myers, D. J.; Kariuki, N. N.; Nowicki, T.; Stamenkovic, V.; Kang, Y.; Li, D.; Papageorgopoulos, D. Best Practices and Testing Protocols for Benchmarking ORR Activities of Fuel Cell Electrocatalysts Using Rotating Disk Electrode. *Electrocatalysis* **2017**, *8* (4), 366–374. https://doi.org/10.1007/s12678-017-0378-6.
